# Immune‐Related Adverse Events and Therapeutic Outcomes After Stopping Immune Checkpoint Inhibitors due to Toxicity Among Patients With Metastatic Melanoma (University Hospitals Sussex)

**DOI:** 10.1002/cam4.72119

**Published:** 2026-07-22

**Authors:** Karmen Iessa, Kavita Kantilal, Ieda Garekyaragh, Lucy Paget, Yvonne Mangan, Yousif A. Shamsaldeen

**Affiliations:** ^1^ Department of Pharmacy, School of Applied Sciences University of Brighton Moulsecoomb, Brighton East Sussex UK; ^2^ University Hospitals Sussex, Royal Sussex County Hospital Brighton East Sussex UK; ^3^ Department of Pharmacy and Biomolecular Sciences, The Faculty of Health, Innovation, Technology and Science (HITS) Liverpool John Moores University Liverpool Merseyside UK

**Keywords:** immune checkpoint inhibitors, immune‐related adverse events, immunotherapy, melanoma

## Abstract

Melanoma is an aggressive type of skin cancer. Immune checkpoint inhibitors (ICIs): pembrolizumab, ipilimumab and/or nivolumab are recommended for the treatment of metastatic melanoma. ICIs enhance the immune response by blocking PD‐1 and/or CTLA‐4, improving remission and overall survival (OS). However, ICIs can cause immune‐related adverse events (irAEs) such as colitis and dermatitis. Recent studies have demonstrated association between irAEs and improved tumour regression. This study aimed to evaluate the relationship between OS and stopping ICIs therapy due to immunological toxicity in metastatic melanoma patients. This study was approved by The University Hospitals Sussex NHS Foundation Trust (UHSussex) (reference 1963) and the University of Brighton (reference 2024–12,878‐Shamsaldeen). Data collected from files of patients diagnosed with metastatic melanoma treated with ICIs at UHSussex between October 2011 to December 2022. In a total of 344 metastatic melanoma patients, the OS was 41.3%. There were 184 patients (53.5%) experienced irAEs. Among 202 patients who died by the cut‐off point, there were 115 (56.9%) patients who did not experience irAE, while 68.3% of the 142 patients who were alive experienced irAE revealing overall association (*p* < 0.001, Pearson's *R* = 0.249) with logistics regression analysis showed association between irAE and OS (*p* < 0.001). Kaplan–Meier survival analysis showed significant longer OS (*p* < 0.001) for patients experienced irAE. Stopping ICIs due to toxicity reported in 83 patients from which 56 patients were alive by the cut‐off point (67.5%) revealing overall association (*p* < 0.001, Pearson's *R* = 0.171) with logistics regression analysis showed association between stopping ICI due to toxicity and OS (*p* < 0.001). Kaplan–Meier survival analysis showed significant longer OS (*p* = 0.027) in patients whom their ICI therapy was stopped due to immune‐related toxicity. In conclusion, the positive correlation between irAEs and survival may highlight the potential value of irAEs and irAE‐related toxicity as biomarkers for therapeutic efficacy in advanced melanoma management.

## 1 Introduction

Melanoma represents a significant public health concern in the United Kingdom where it is ranking as the fifth most common type of cancer and accounting for 5% of all new cancer diagnoses [1, 2]. With approximately 17,500 new cases annually, melanoma incidence has increased dramatically by 147% between 1993 and 2019. Projections indicate a further 9% rise between 2023–2025 and 2038–2040, with annual cases expected to reach 26,500, highlighting the growing burden of this malignancy [3].

The development of immune checkpoint inhibitors (ICIs), such as pembrolizumab, nivolumab and ipilimumab, has enhanced the treatment of metastatic melanoma. These monoclonal antibodies are recommended by The National Institute for Health and Care Excellence (NICE) for patients with unresectable stage III or untreated stage IV melanoma. Treatment decisions are based on the patient's overall health, co‐morbidities, tumour biology, presence of symptomatic brain metastases and whether the combination ICI toxicity will be tolerated. Shared decision making is encouraged after an assessment of all the risks and benefits of treatment. The option of single agent ICI with pembrolizumab or nivolumab is offered if the combination of ipilimumab and nivolumab is deemed unsuitable based on the assessment and patient's goals and preferences. Novel therapies, such as Opdualag, a combination of relatlimab and nivolumab for untreated advanced melanoma, have also been approved by NICE in February 2024 [1, 4].

Ipilimumab targets cytotoxic T‐lymphocyte antigen 4 (CTLA‐4) on the surface of T‐cells. CTLA‐4 is an inhibitory receptor that downregulates T‐cell activation by binding to CD80 and CD86 proteins on antigen‐presenting cells (APCs). By blocking this interaction, ipilimumab reduces the inhibitory signals that limit T‐cell proliferation, thereby enhancing T‐cell activation and the anti‐tumour immune response [5]. Nivolumab and pembrolizumab, on the other hand, target the programmed death protein‐1 (PD‐1) receptor, which when bound by programmed death ligand 2 (PD‐L2) leads to limiting T‐cell activity towards cancer cells. By inhibiting the PD‐1 pathway, nivolumab and pembrolizumab block the formation of this inhibitory complex, allowing for sustained T‐cell activation and an enhanced anti‐tumour immune response [6, 7]. A population‐based study with a minimum follow‐up of 6.5 years on metastatic or unresectable melanoma showed the median OS was 72.1 months with nivolumab and ipilimumab combination therapy, 36.9 months with nivolumab and 19.9 months with ipilimumab‐treated group [8]. Moreover, longer follow‐up data has demonstrated pembrolizumab's superior efficacy, with 34.0% of advanced melanoma patients surviving at 10 years, compared to 23.6% with ipilimumab treatment, highlighting a significant advancement in therapeutic options [9].

Immune‐related adverse events (irAEs) present significant challenges in ICIs therapy. CTLA‐4 inhibitors predominantly cause colitis, rash, hypophysitis and pruritus, while PD‐1 inhibitors more commonly induce pneumonitis, hypothyroidism, arthralgia, myalgia and vitiligo [10]. Notably, combination therapy using anti‐PD‐1 and anti‐CTLA‐4 agents demonstrates the highest overall incidence at 59%–72% [11], underscoring increased risk of irAEs with dual checkpoint blockade. Thus, the combination therapy particularly affects the gastrointestinal (GI) system, with an incidence rate of 44% compared to 23% for anti‐CTLA‐4 monotherapy and < 20% for anti‐PD‐1 therapy. Additional adverse events include hepatitis, autoimmune endocrinopathies, neurologic dysfunction and immune‐mediated pneumonitis [12]. Management strategies will depend on the severity of the irAE which are graded according to the Common Terminology Criteria for Adverse Events (CTCAE) [13]. Grade 1 (mild) irAEs typically require monitoring without intervention. Grade 2 (moderate) may necessitate temporary ICI cessation and possible glucocorticoid administration. Grade 3/4 (severe) irAEs require immediate hospitalisation, prompt immunosuppressive corticosteroid treatment and potential ICI discontinuation. For steroid‐refractory cases, specific agents like mycophenolate mofetil may be utilised for hepatitis, and infliximab for colitis to control inflammation cascades [12, 14, 15, 16, 17].

The association between irAEs and immunotherapy effectiveness reveals complex immunological interactions. Meta‐analyses have demonstrated correlations between irAEs and clinical outcomes across various cancer types [18, 19]. Multiple studies have investigated specific irAE types and their relationship to treatment outcomes. A comprehensive meta‐analysis of 30 trials revealed that patients with irAEs experienced overall survival (OS) benefits (HR 0.54; 95% CI, 0.45–0.65; *p* < 0.001) [20]. Notably, specific irAE types demonstrated favourable associations with improved treatment outcomes where endocrine irAEs: HR 0.52 (95% CI, 0.44–0.62; *p* < 0.001), dermatological irAEs: HR 0.45 (95% CI, 0.35–0.59; *p* < 0.001) and GI‐irAEs: HR 0.68 (95% CI, 0.51–0.89; *p* = 0.005) [21], thus serving as a potential clinical biomarker for ICI response [22]. In patients with melanoma undergoing anti‐CTLA‐4 therapy, this association appears particularly pronounced, with severe (Grade ≥ 3) irAEs being correlated with improved therapeutic outcomes [22, 23]. The underlying immunological mechanisms connecting irAEs to treatment efficacy continue to require further investigation.

Accordingly, this study aims to evaluate the impact of ICIs on melanoma patients who received ICI for unresectable or advanced disease at University Hospitals Sussex NHS Foundation Trust (UHSussex). The study seeks to explore the relationship between irAEs occurrence and patients' survival, with particular focus on stopping ICIs therapy due to immunological toxicity in metastatic melanoma patients receiving ICIs.

## 2 Methods

### 2.1 Ethical Approval

This study was approved at UHSussex (reference 1963) followed by approval at the University of Brighton (reference 2024‐12878‐Shamsaldeen). Identifiable patient information was anonymised and saved to a secure University of Brighton OneDrive platform. Since the study involved retrospective data collection of unidentifiable patients' information from medical records without direct patients participation nor biological materials collection, therefore, consents from patients were not needed and therefore, consent was exempt by the ethics committees for this purpose.

### 2.2 Study Design and Population

Electronic health records were retrospectively reviewed between October 2011 and December 2022 to identify patients treated with ICIs for unresectable or advanced malignant melanoma at UHSussex. Demographic information, BRAF status, irAEs and treatment outcomes were recorded. Treatment outcomes included treatment discontinuation, hospitalisation and OS. OS was defined as the time from the beginning of immunotherapy to death from any cause, censored at the date of last follow‐up [24]. For the purposes of the study, the data cutoff on 22 December 2022 was selected.

### 2.3 Statistical Analysis

Statistical analysis was performed using SPSS software (version 29.0.2.0). The chi‐squared (*χ* ^2^) test was applied through *p* value, with 95% confidence intervals (CIs) to determine the relationship between two categorical variables, and whether observed differences in results are likely due to chance. Moreover, Pearson correlation coefficient (PCC) with *R* value was calculated to determine the correlation coefficient that measures linear correlation between two sets of data. To analyse OS, the Kaplan–Meier method was used to estimate the median survival with 95% CI and Cox model to estimate hazard ratios (HRs) throughout groups' comparison with 95% CI. To assess IrAEs as well as stopping therapy association with objective response rate, we used logistic regression analysis to estimate odds ratios (ORs) with 95% CIs throughout the group comparisons [24].

## 3 Results

A total of 427 patients diagnosed with malignant melanoma treated with ICIs were identified. There were 344 patients who received ICI for unresectable or advanced disease (in the metastatic setting).

Among the 344 patients, 191 (55.5%) were male. The average age of the patients at the cut‐off point was 70 years old, with the youngest patient aged 26 years old and the oldest was 96 years old. Moreover, the mean age at start of ICI was 66 (age range 24–90) years. The age range of 70s was the highest with 34.9% of the patients. BRAF mutation was recorded for 35.1% (121) patients (Table 1). Over 80% (276) patients received ICIs as first line therapy. Of these, 64.9% (179) patients received single agent ICI (with 66.5%, 17.9% and 15.6% receiving pembrolizumab, nivolumab, and ipilimumab respectively) with a mean age of 72 years. Ninety‐seven (35.1%) patients received combination ipilimumab and nivolumab in the first line setting, with a mean age of 58 years. Among patients receiving second line ICI therapy, 21.5% [14] received combination ipilimumab and nivolumab, with a mean age of 58 years. Among the 78.5% (51) patients who received single agent ICI, approximately half of patients (30, 46.2%) were treated with ipilimumab, a CTLA‐4 blocker. The mean age of patients receiving single agent ICI in the second line setting was 65 years. Three patients received ipilimumab in the third line setting with a mean age of 66 years.

**TABLE 1:** Characteristics of melanoma patients who received ICI for unresectable or advanced disease in University Hospitals Sussex NHS Foundation Trust (UHSussex) from the period between October 2011 and December 2022.

Variable	*N* (%)
Sex	
Male	191 (55.5%)
Female	153 (44.5%)
Age range (24–90)	
20–29	1 (0.3%)
30–39	10 (2.9%)
40–49	13 (3.8%)
50–59	41 (11.9%)
60–69	67 (19.5%)
70–79	120 (34.9%)
80–89	81 (23.5%)
90–99	11 (3.2%)
BRAF status	
Wild type	217 (63.1%)
Mutation	121 (35.2%)
Not recorded	6 (1.7%)

At data cutoff 142 treated patients were still alive or continuing with ICI therapy. The combination regimen of ipilimumab and nivolumab showed the highest OS with 52.3%, while ipilimumab monotherapy was with the least OS (14.8%) as shown in Table 2. Moreover, analysis of sex‐stratification of survival data in 344 melanoma metastatic patients revealed 40.3% of male patients and 42.5% of female patients were alive at the cut‐off date with the highest percentage of alive patients were in pembrolizumab monotherapy: 62% in male and 51.6% in female patients.

**TABLE 2:** Overall survival at cutoff point of December 2024 in melanoma patients who received ICI for unresectable or advanced disease in University Hospitals Sussex NHS Foundation Trust (UHSussex) from the period between October 2011 and December 2022.

	Total no. treated	Total no. alive at data cutoff	Overall survival rate (%)
All ICI	344	142	41.3%
Ipilimumab and Nivolumab	111	58	52.3%
Pembrolizumab	135	60	44.4%
Nivolumab	37	15	40.5%
Ipilimumab	61	9	14.8%

**FIGURE 1:**
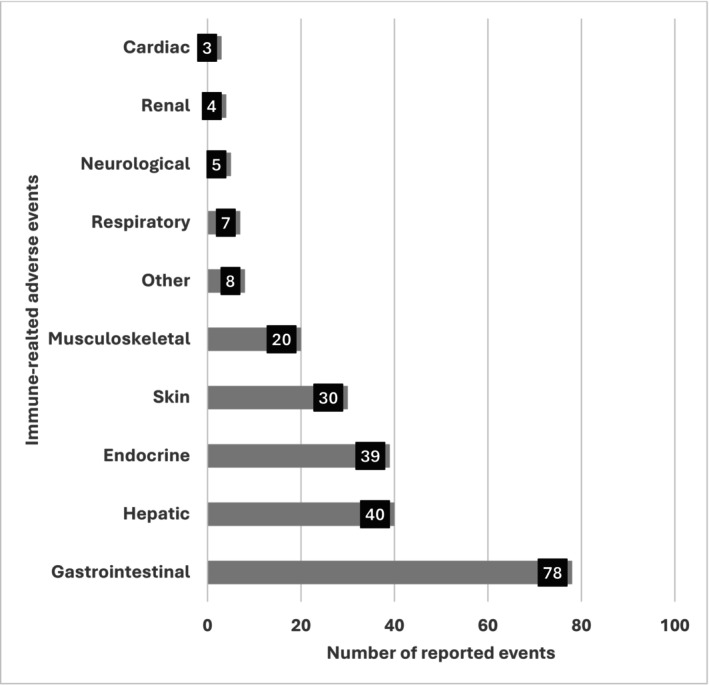
The prevalence of the different organs affected by the irAEs in 344 melanoma patients who received ICI for unresectable or advanced disease in University Hospitals Sussex NHS Foundation Trust (UHSussex) from the period between October 2011 and December 2022.

Among the 344 melanoma metastasis patients, there were 184 patients (53.5%) who experienced any grade of irAEs, from which there were 56 melanoma metastasis patients (30.4% of total patients with irAEs) who experienced grade 3 or greater irAEs during ICI therapy. Analysis of sex‐stratification of irAEs data in 344 melanoma metastatic patients revealed 52.9% of male patients and 54.2% of female patients experienced irAEs, with the most irAEs reported in combination therapy, with 74.2% in male patients and 71.4% in female patients. The top five organs affected by irAEs included the GI, hepatic, endocrine, skin and musculoskeletal (Figure 1 and Table S1). From the melanoma metastasis patients, there were 83 patients (24.3%) who discontinued ICI therapy due to irAEs.

**FIGURE 2:**
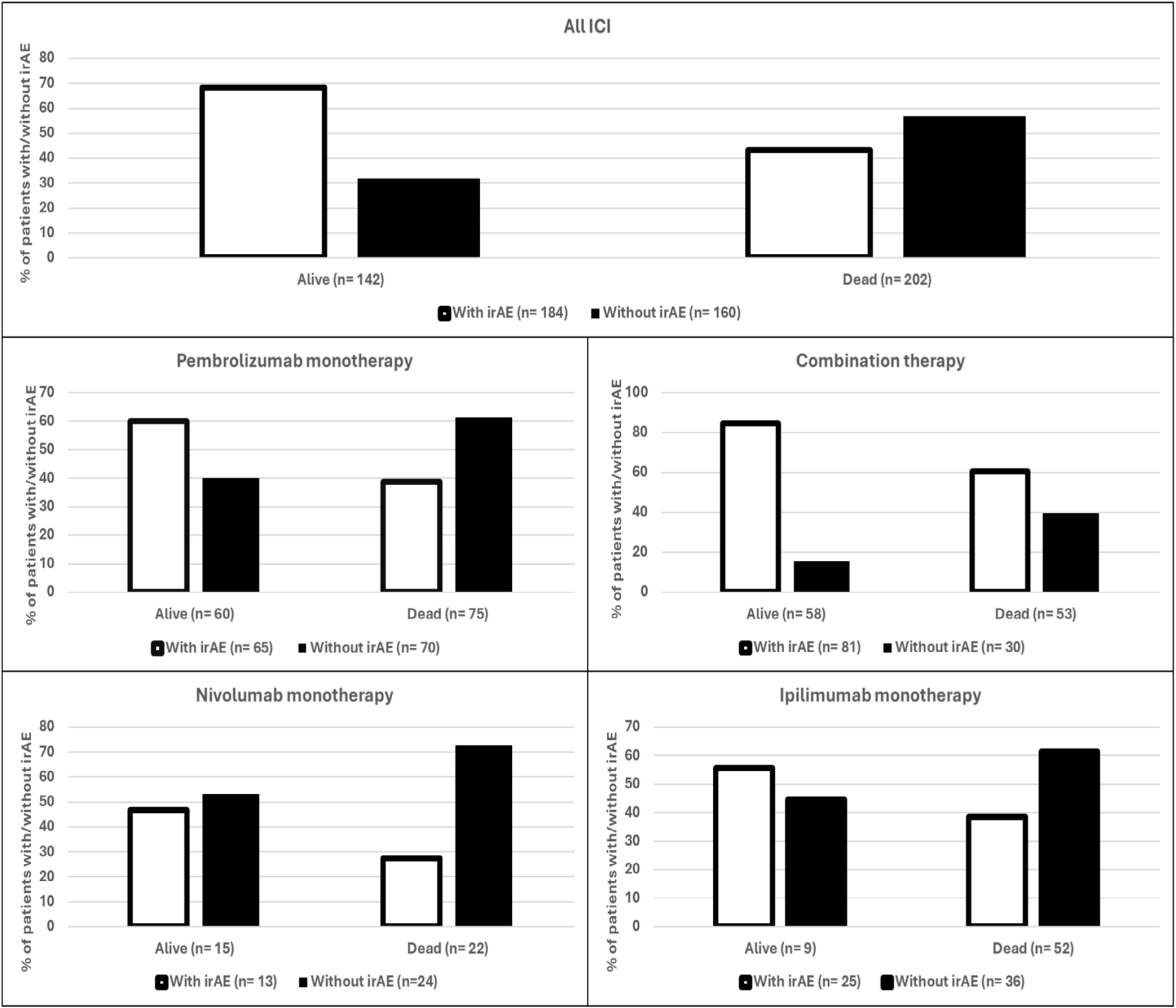
Association between immune‐related adverse events (irAEs) and patients' survival in 344 melanoma patients who received immune checkpoint inhibitors (ICI) for unresectable or advanced disease in University Hospitals Sussex NHS Foundation Trust (UHSussex). All ICI: All 344 melanoma patients who received any ICI. Combination therapy: Ipilimumab and nivolumab. All ICI: 68.3% of patients who were alive showed irAE, while 56.9% of dead patients did not show irAE (*p* < 0.001, Pearson's *R* = 0.249). Pembrolizumab monotherapy: 60% of patients who were alive showed irAE, while 61.3% of dead patients did not show irAE (*p* = 0.016, Pearson's *R* = 0.212). Combination therapy of ipilimumab and nivolumab: 84.5% of patients who were alive showed irAE, while 39.6% of dead patients did not show irAE (*p* = 0.005, Pearson's *R* = 0.271). Nivolumab monotherapy: 64.7% of patients who were alive showed irAE, while 72.7% of dead patients did not show irAE (*p* = 0.3, Pearson's *R* = 0.199). Ipilimumab monotherapy: 55.6% of patients who were alive showed irAE, while 61.57% of dead patients did not show irAE (*p* = 0.467, Pearson's *R* = 0.123).

**FIGURE 3:**
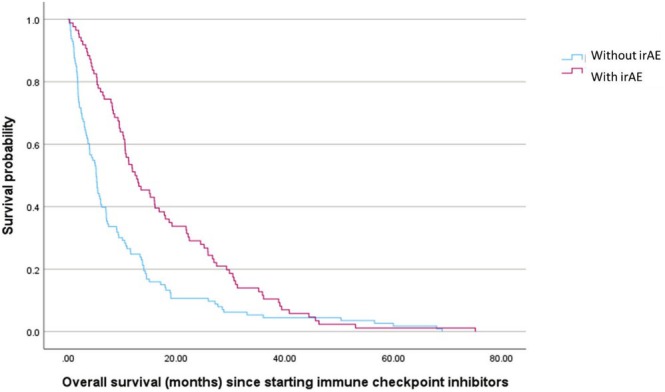
Overall survival (OS) with immune checkpoint inhibitors depending on the incidence of immune‐related adverse events (irAEs) in 344 melanoma patients who received immune checkpoint inhibitors (ICI) for unresectable or advanced disease in University Hospitals Sussex NHS Foundation Trust (UHSussex). The median OS was significantly longer for patients experiencing IrAEs with mean difference 9.8 months without irAEs compared to 17.4 months for patients experience irAEs (*p* < 0.001).

The overall analysis of the 344 melanoma metastatic patients demonstrated an association between irAEs and patients' survival. Among the 202 patients who died by the cut‐off point, there were 115 patients who did not experience irAE contributing to 56.9%. In contrast, 68.3% of the 142 melanoma metastasis patients who were alive experienced irAE revealing an overall good association (*p* < 0.001, Pearson's *R* = 0.249). Among the utilised ICIs, the best outcomes were in both the combination regimen (*p* = 0.004, Pearson's *R* = 0.271) and pembrolizumab monotherapy (*p* = 0.013, Pearson's *R* = 0.212). Thus, among the 111 melanoma metastasis patients who were treated with the combination regimen, there were 58 patients who were alive, of which 84.5% of them (*n* = 49) experienced irAE. Moreover, when looking through the irAE categorisation, there were a total of 81 melanoma metastasis patients who were treated with the combination therapy, of which 60.5% of them were alive at the cut point, while 30% of the patients who did not experience irAE (9/30) were alive. Similarly, among the 135 melanoma metastasis patients who were treated with pembrolizumab monotherapy, there were 60 patients who were alive, of which 60% of them (*n* = 36) experienced irAE as shown in Figure 2. Moreover, when looking through the irAE categorisation, there were a total of 65 melanoma metastasis patients who were treated with the pembrolizumab monotherapy, of which 55.4% of them were alive at the cut point, while 34.3% of the patients who did not experience irAE (24/70) were alive. When analysing the OS through Kaplan–Meier, the median OS was significantly longer (*p* < 0.001) for patients experiencing IrAEs with a mean difference of 9.8 months without irAEs compared to 17.4 months for patients experiencing irAEs (HR, 0.565; 95% CI, 0.425–0.752; *p* < 0.001) as shown in Figure 3. Moreover, to assess irAE association with OS, logistic regression analysis showed ORs = 0.351 with 95% CI, 0.224–0.550; *p* < 0.001.

**FIGURE 4:**
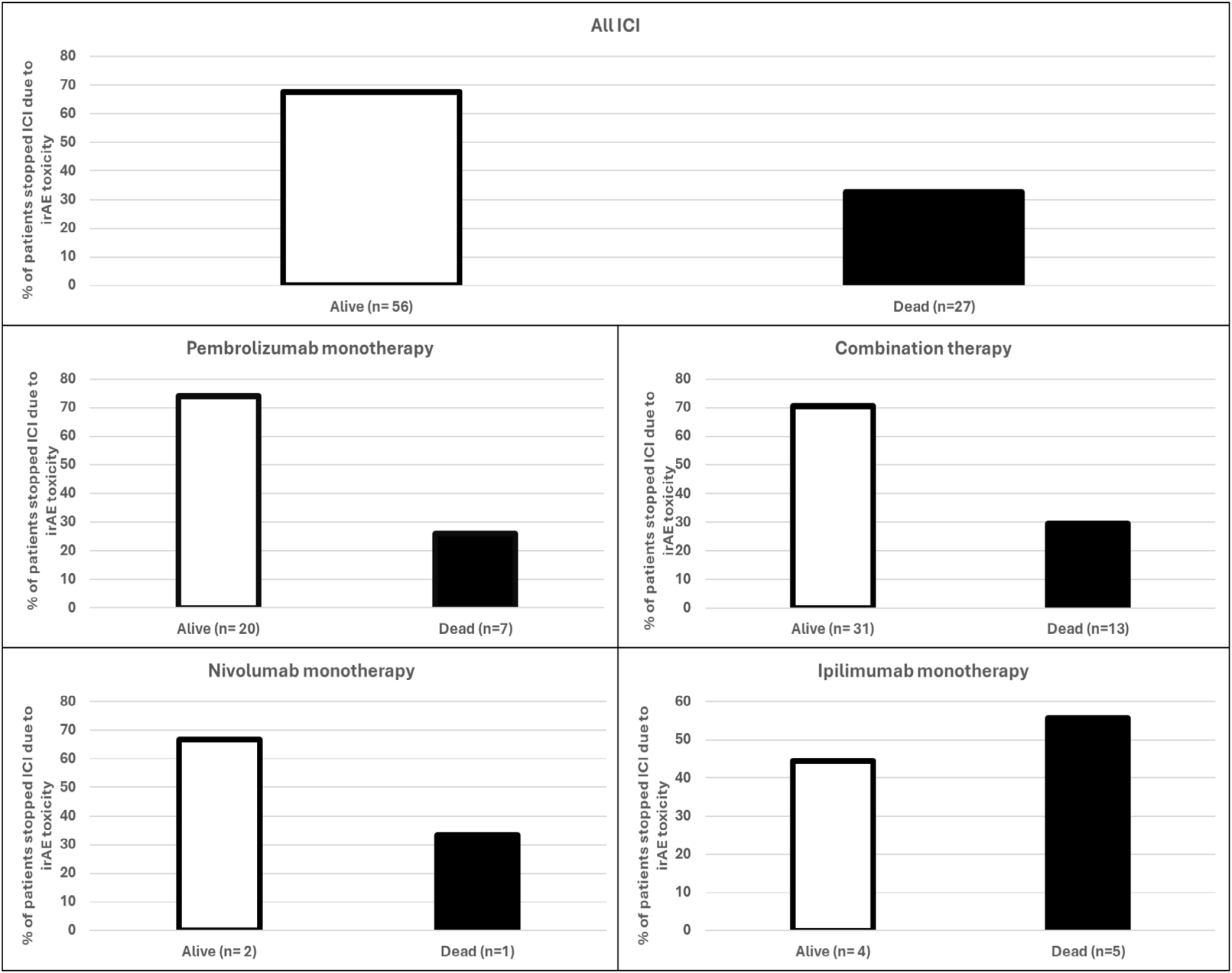
Association between stopping therapy due to immune‐related toxicity and patients' survival in 344 melanoma patients who received immune checkpoint inhibitors (ICI) for unresectable or advanced disease in University Hospitals Sussex NHS Foundation Trust (UHSussex). All ICI: All 344 melanoma patients who received any ICI. Combination therapy: Ipilimumab and nivolumab. All ICI: 67.5% of patients whose therapy was stopped due to toxicity were alive (*p* < 0.001, Pearson's *R* = 0.171). Pembrolizumab monotherapy: 74.1% of patients whose therapy was stopped due to toxicity were alive (*p* < 0.001, Pearson's *R* = 0.279). Combination therapy of ipilimumab and nivolumab: 70.5% of patients whose therapy was stopped due to toxicity were alive (*p* < 0.001, Pearson's *R* = 0.133). Nivolumab monotherapy: 33.3% of patients whose therapy was stopped due to toxicity were alive (*p* = 0.005, Pearson's *R* = 0.352). Ipilimumab monotherapy: 44.4% of patients whose therapy was stopped due to toxicity were alive (*p* = 0.019, Pearson's *R* = 0.009).

The overall analysis of the 344 melanoma metastatic patients demonstrated association between discontinuation of ICI due to irAE‐related toxicity and patients' survival. Thus, the overall incidents of stopping the therapy due to toxicity were 83 patients (24.13%). Among the total 83 patients who their ICIs therapy was stopped due to toxicity, there were a total of 56 patients who were alive by the cut‐off point (67.5%) revealing an overall good association (*p* < 0.001, Pearson's *R* = 0.171). Colitis was the most common irAEs associated with stopping therapy in metastatic melanoma patients with 42 cases from which 14 patients were dead while 28 patients were alive (*p* = 0.876, Pearson's *R* = −0.17). Among the utilised ICIs, the best outcomes were in both the combination regimen (*p* < 0.001, Pearson's *R* = 0.133) and pembrolizumab monotherapy (*p* < 0.001, Pearson's *R* = 0.279). Thus, among the 111 melanoma metastasis patients who were treated with the combination regimen, there were 44 patients whom their ICI therapy was stopped due to irAE‐related toxicity, from which 70.5% of them were alive at the cut‐off point. Similarly, Thus, among the 135 melanoma metastasis patients who were treated with pembrolizumab monotherapy, there were 27 patients whom their ICI therapy was stopped due to irAE‐related toxicity, from which 74.1% of them showed survival Figure 4. In contrast, after stopping ICI therapy due to toxicity, 27 patients died at the cut‐off points, from which 20 patients had their full tracking data available to know the period through which they lived despite stopping the therapy. Thus, there were six patients (30%) who were alive for up to 6 months, while 20% of patients lived for up to 12 months, and 25% were alive for up to 24 months in addition to two patients survived for up to 3 years, one patient (5%) lived for up to 42 months and another patient lived for 4 years, while there was one patients who lived for up to 5 years.

**FIGURE 5:**
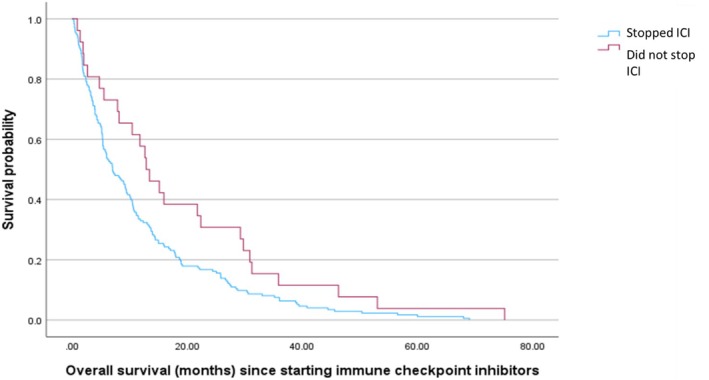
Overall survival (OS) with immune checkpoint inhibitors depending on the incidence of immune‐related toxicity and stopping therapy in 344 melanoma patients who received immune checkpoint inhibitors (ICI) for unresectable or advanced disease in University Hospitals Sussex NHS Foundation Trust (UHSussex). The median OS was significantly longer for patients who experienced immune‐related toxicity and their immune checkpoint inhibitors therapy was stopped with mean difference 12.2 months in patients who did not experience immune‐related toxicity and stopping therapy compared to 19.4 months for patients experienced immune‐related toxicity and stopping therapy (*p* = 0.027).

**FIGURE 6:**
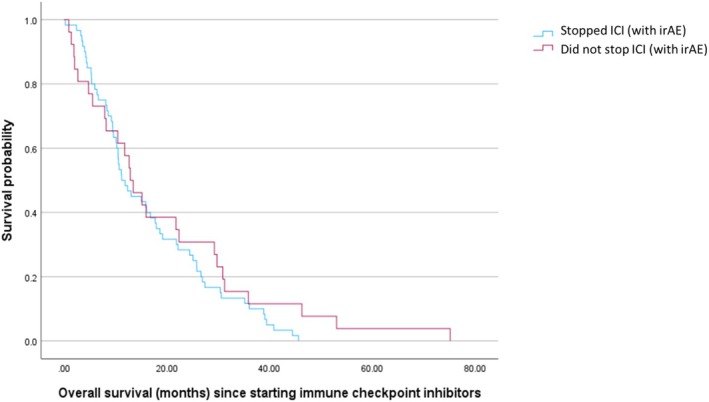
Overall survival (OS) with immune checkpoint inhibitors depending on the incidence of immune‐related toxicity and stopping therapy in patients who developed immune‐related adverse events (irAEs) in 184 melanoma patients who experienced IrAEs when received immune checkpoint inhibitors (ICI) for unresectable or advanced disease in University Hospitals Sussex NHS Foundation Trust (UHSussex). The median OS was not significantly different (*p* = 0.285) with mean survival = 19.4 months for patients who developed immune‐related toxicity and requiring ICI stop versus 16.6 months for patients who developed irAEs without immune‐related toxicity and not requiring ICI stop.

When analysing the OS through Kaplan–Meier, the median OS was significantly longer (*p* = 0.027) for patients whom their ICI therapy was stopped due to immune‐related toxicity with mean difference of 12.2 months for patients who did not stop their therapy immune‐related toxicity compared to 19.4 months for patients experienced immune‐related toxicity and their ICI therapy was stopped (HR, 0.623; 95% CI, 0.409–0.951; *p* = 0.028) as shown in Figure 5. Moreover, to assess developing immune‐related toxicity and requiring stopping their ICI therapy association with OS, logistics regression analysis showed ORs = 0.237 with 95% CI of 0.140–0.401; *p* < 0.001. However, when patients with irAEs only were studied based on their status of developing immune‐related toxicity and requiring stopping their ICI therapy, there was not significant difference (*p* = 0.285) in the rate of survival when compared with patients developed irAEs without immune‐related toxicity (mean survival = 19.4 months for patients developed immune‐related toxicity and requiring ICI stop vs. 16.6 months for patients developed irAEs without immune‐related toxicity and not requiring ICI stop) (HR, 0.768; 95% CI, 0.472–0.1.248; *p* = 0.286) as shown in Figure 6. When assessing developing immune‐related toxicity and requiring stopping their ICI therapy association with OS in patients who experienced irAEs, logistics regression analysis showed ORs = 0.329 with 95% CI of 0.180–0.605; *p* < 0.001.

## 4 Discussion

This comprehensive investigation examined the multifaceted associations between irAEs and clinical outcomes in melanoma patients who received ICI for unresectable or advanced disease. Employing a rigorous retrospective methodology, we systematically analysed the intricate relationships between irAEs and survival. Our analysis transcended conventional clinical assessments by stratifying outcomes across multiple clinically relevant dimensions, including patient demographic characteristics such as sex and molecular genomic profiles defined by BRAF mutation status.

Our study provides compelling evidence of the nuanced relationship between irAEs and treatment efficacy in advanced melanoma patients. As shown in Table 2, the OS in all patients receiving any ICIs therapy was 41.3%, with combination therapy of ipilimumab and nivolumab showing OS of 52.3%, while the pembrolizumab treatment group had an OS of 44.4%, with slightly less OS (40.5%) in the nivolumab‐treated group. However, the ipilimumab‐only treatment group showed the lowest OS (14.8%). The pivotal Phase 3 KEYNOTE‐006 trial provides further compelling evidence for the superiority of pembrolizumab over ipilimumab in advanced melanoma, with improved progression‐free survival, OS and overall response rates. 5‐year OS was 38.7% (95% CI 34.2–43.1) in the pembrolizumab‐treated patients and 31.0% (25.3–36.9) in the ipilimumab group [25]. As shown in Table 3, irAEs were reported in 53.5% of the melanoma patients receiving ICIs for unresectable or advanced disease. This is supported by a recent study that showed the incidence of any‐grade and irAEs in melanoma patients treated with ICI therapy was 60% [26]. The most common irAE was GI‐irAEs, followed by hepatic, endocrine, dermatologic and musculoskeletal irAEs (Figure 1). These common top five irAEs are similar to what has been reported in melanoma patients treated with ICIs [27, 28].

**TABLE 3:** Immune related adverse events by treatment regimen in melanoma patients who received ICI for unresectable or advanced disease in University Hospitals Sussex NHS Foundation Trust (UHSussex) from the period between October 2011 and December 2022.

	No. of patients treated with ICI	No. of patients with any grade irAEs (%)	No. of patients with Grade 3 or more irAEs (% to irAEs)
All ICI	344	184 (53.5%)	56 (30.4%)
Ipilimumab and Nivolumab	111	81 (73.0%)	38 (46.9%)
Pembrolizumab	135	65 (48.1%)	8 (12.3%)
Nivolumab	37	13 (35.1%)	2 (15.4%)
Ipilimumab	61	25 (41.0%)	8 (32%)

The association between irAEs and OS was investigated and it showed there was an association between irAE occurrence and clinical outcomes (*p* < 0.001, Pearson's *R* = 0.249) (Figure 2). Such OS was supported by Kaplan–Meier analysis that showed the median OS was significantly longer (*p* < 0.001) for patients experiencing irAEs shown in Figure 3. This observation suggests a potential association between irAEs and therapeutic efficacy. Furthermore, this finding aligns with emerging literature suggesting irAEs may serve as potential immunological biomarkers of treatment efficacy. These observations complement existing meta‐analytical evidence indicating that specific irAE types—particularly endocrine, dermatological and GI—correlate with improved treatment outcomes [20]. The mechanism potentially relates to sustained immune activation and minimal treatment interruption, though the precise immunological pathways remain incompletely understood. Our findings suggest that irAEs, contrary to being solely adverse events, may represent dynamic markers of immunological engagement. The differential outcomes across irAE types and severities underscore the complexity of ICI responses, highlighting the need for personalised treatment approaches and continued investigation into the immunological mechanisms underlying these observations. Pembrolizumab monotherapy (*p* = 0.013, Pearson's *R* = 0.212) and the combination of ipilimumab and nivolumab (*p* = 0.004, Pearson's *R* = 0.271) showed the highest correlation between irAEs and survival. Thus, there were 60 patients alive among the 135 patients who were treated with pembrolizumab monotherapy for unresectable or advanced disease, while 58/111 of melanoma metastasis patients were alive at the cut‐off point when treated with the combination regimen (Figure 2). Immune checkpoint blockade therapy compromises self‐tolerance by interfering with regulatory pathways, triggering an immune response that simultaneously attacks cancerous and normal cells. This dual activity manifests clinically as both therapeutic efficacy and irAEs, potentially explaining why patients experiencing these adverse events demonstrate better clinical responses [29]. Stratified analysis demonstrated a consistent association between irAEs and survival regardless of patient sex. The sex‐independent relationship between irAEs and survival aligns with the established biological mechanisms of ICI therapy. Established research demonstrates immunological sex differences, with females mounting stronger immune responses that enhance pathogen clearance but increase autoimmune susceptibility [30]. Further investigations were conducted to examine the association and correlation between survival and severe irAE that required immediate stoppage of ICI therapy due to toxicity. As shown in Figure 4, there were 83 patients whose ICI therapy was stopped due to toxicity (24.13%). Among the 83 patients, there were a total of 56 patients who were alive by the cut‐off point (67.5%) revealing an overall positive association (*p* < 0.001, Pearson's *R* = 0.171). In ipilimumab monotherapy, there were 9 (14.7%) patients suffering from toxicity requiring therapy stoppage, from which only 4 patients were alive at the cut‐off point. In contrast, only 3 patients (8.1%) had their therapies stopped due to toxicity in the nivolumab treatment group. Moreover, the combination therapy of ipilimumab and nivolumab showed 40% of patients stopping their therapy due to toxicity (44 patients), from which 31 patients (54.4%) were alive at the cut‐off point. Additionally, in pembrolizumab monotherapy, 20.3% of patients stopped their therapy due to toxicity (27 patients), from which 20 patients (74.1%) were alive at the cut‐off point. It should be noted that the majority of irAEs in the melanoma metastatic patients who had their therapy stopped due to toxicity were ≥ Grade 3 (53.7% Grade 3 and 9.0% Grade 4) with three patients of Grade 1 irAEs and 22 patients of Grade‐2 (32.8%). However, among the 22 patients of Grade 2 irAE, there were 13 patients who required corticosteroid treatments to control their irAEs (59.1%) revealing potential serious irAEs that can be classified at a higher grade based on other factors such as the affected organ, the presence of other irAEs and age. Thus, among the 22 patients who experienced Grade 2 irAE and stopped ICI due to toxicity, there were 12 patients who experienced irAEs in more than one organ system including musculoskeletal together with skin and GI. Moreover, there were two patients who experienced one irAE but in the liver and hence causing the immediate stoppage of the ICIs. However, there were eight patients who experienced irAE in the GI system only, revealing the possibility of either underscoring the irAE, patients' decision to stop the therapy, or the contribution of other co‐morbidities that make the decision to stop the ICI tailored to the patient's case not only the grade of the irAE. Similarly, among the three patients who experienced irAE of Grade 1, there were 2 patients with hepatic irAE and one with colitis.

Among the utilised ICIs, the best outcomes were in both the combination regimen (*p* < 0.001, Pearson's *R* = 0.133) and pembrolizumab monotherapy (*p* < 0.001, Pearson's *R* = 0.279). Thus, among the 111 melanoma metastasis patients who were treated with the combination regimen, there were 44 patients whose ICI therapy was stopped due to irAE‐related toxicity, from which 70.5% of them were alive at the cut‐off point. Similarly, among the 135 melanoma metastasis patients who were treated with pembrolizumab monotherapy, there were 27 patients whose ICI therapy was stopped due to irAE‐related toxicity, from which 74.1% of them were alive at the cut‐off point (Figure 4). This was supported by Kaplan–Meier analysis that showed a significant increase in median OS (*p* = 0.027) for patients whose ICI therapy was stopped due to immune‐related toxicity (Figure 5). This positive correlation between irAEs and treatment response supports the hypothesis that both therapeutic effects and adverse events stem from a common mechanism of enhanced immune system activation, with the severity of immune‐related toxicity potentially reflecting the degree of immune reinvigoration [12]. In metastatic disease, where tumour burden is higher, the development of irAEs may reflect robust immune activation necessary for effective anti‐tumour responses, thereby serving as a potential biomarker for treatment efficacy. The significance of context‐specific approaches to evaluating and controlling immune‐related toxicities in melanoma immunotherapy is highlighted by our findings, which also indicate the potential prognostic relevance of irAEs specifically in the context of metastatic illness. Moreover, the continuity of the ICI effect despite being stopped due to toxicity was tracked in 20 out of 27 patients who died after stopping the therapy with an average of 19 months survival after stopping the therapy, revealing potential continuous effect despite stopping the therapy. These findings are supported by a similar study conducted in colorectal cancer patients treated with ICI [31].

This study has several limitations. As this study's outcomes are from real‐world cases, therefore, under‐reporting of irAEs is highly likely. Thus, toxicities may potentially be underreported in the early years due to limited recognition of irAEs, which may have contributed to delays in management. Even when irAEs are managed appropriately, decisions to resume ICI therapy remain complex. Factors such as tumour response, treatment duration, intensity and duration of immunosuppression, time to irAE resolution, patient performance status, patient preferences and treatment goals were considered. These decisions should be individualised within a multidisciplinary team. Moreover, toxicity breakdown was not included as many patients presented with multiple organ toxicities of varying grades. Thus, we did not collect data on whether these irAEs occurred simultaneously or sequentially, nor did we capture specific reasons for discontinuation. Therefore, the data lack the exact date of when irAEs were developed and therefore, landmark analysis was not conducted to investigate time‐to‐event analysis. Additional limitation includes the change in guidelines managing metastatic melanoma. Thus, in the metastatic patients that we analysed. First line immunotherapy *n* = 276. Second‐ and third‐line immunotherapy *n* = 68, of these 21 patients received chemotherapy in the first/s line setting before receiving immunotherapy. As melanoma practice has changed, currently patients are considered for immunotherapy unless it is contraindicated. This may require future studies to investigate the patients based on the line of therapies and the relationship between irAEs and survival. Also, this study focused on the relationship between irAEs and survival without considering other survival cofounding factors such as melanoma disease burden, comorbidities, treatment modalities, age, or gender which are worth investigating in larger studies for generalisable conclusions. Additionally, this study was conducted in University Hospitals Sussex where most patients were from English white ethnicity. Therefore, the conclusion may only be applied to the study's population.

Accordingly, future studies on wider and diverse population focusing on irAEs and remission and survival may provide better detailed data, especially regarding low‐grade irAEs. Moreover, landmark analysis can be conducted to investigate time‐to‐event analysis through Cox models adjusting for time to toxicity and including time‐dependent covariates accounting for irAEs onset.

## 5 Conclusion

In conclusion, this comprehensive study of advanced melanoma patients treated with ICIs at UHSussex demonstrates a robust association between irAEs and survival, particularly in severe toxicity where ICI therapy is stopped. These findings highlight the complex immunological dynamics of ICI therapy and underscore the potential prognostic value of irAEs in guiding personalised treatment approaches for melanoma patients. The optimistic prognosis for the majority of patients should reassure both patients and providers in making the decision to stop immunotherapy.

## Author Contributions

**Ieda Garekyaragh:** investigation, formal analysis. **Kavita Kantilal:** investigation, writing – original draft, formal analysis, supervision. **Yvonne Mangan:** investigation. **Yousif A. Shamsaldeen:** supervision, data curation, formal analysis, project administration, writing – review and editing, validation, methodology, investigation, writing – original draft, resources, software, funding acquisition. **Lucy Paget:** formal analysis, investigation. **Karmen Iessa:** investigation, writing – original draft, formal analysis.

## Funding

The authors have nothing to report.

## Conflicts of Interest

The authors declare no conflicts of interest.

## Supporting information


**Table S1:** Summary of grade distribution, organ‐specific toxicities, steroid requirement, hospitalisation and discontinuation rates in 344 metastatic melanoma patients received ICI for unresectable or advanced disease in University Hospitals Sussex NHS Foundation Trust (UHSussex) from the period between October 2011 and December 2022.

## Data Availability

The data that support the findings of this study are available from the corresponding author upon reasonable request.
