# Correction to “Immune‐Related Adverse Events and Therapeutic Outcomes After Stopping Immune Checkpoint Inhibitors due to Toxicity Among Patients With Metastatic Melanoma (University Hospitals Sussex)”

**DOI:** 10.1002/cam4.72412

**Published:** 2026-10-16

K. Iessa , K. Kantilal , I. Garekyaragh , L. Paget , Y. Mangan , and Y. A. Shamsaldeen , “Immune‐Related Adverse Events and Therapeutic Outcomes After Stopping Immune Checkpoint Inhibitors due to Toxicity Among Patients With Metastatic Melanoma (University Hospitals Sussex),” Cancer Medicine 15, no. 7 (2026): e72119, 10.1002/cam4.72119.PMC1338960442482656

In the originally published article, the legend keys in Figures 5 and 6 were inadvertently reversed. The corrected legends are as follows:

The correct Figures 5 and 6 are provided below.

**FIGURE 5:**
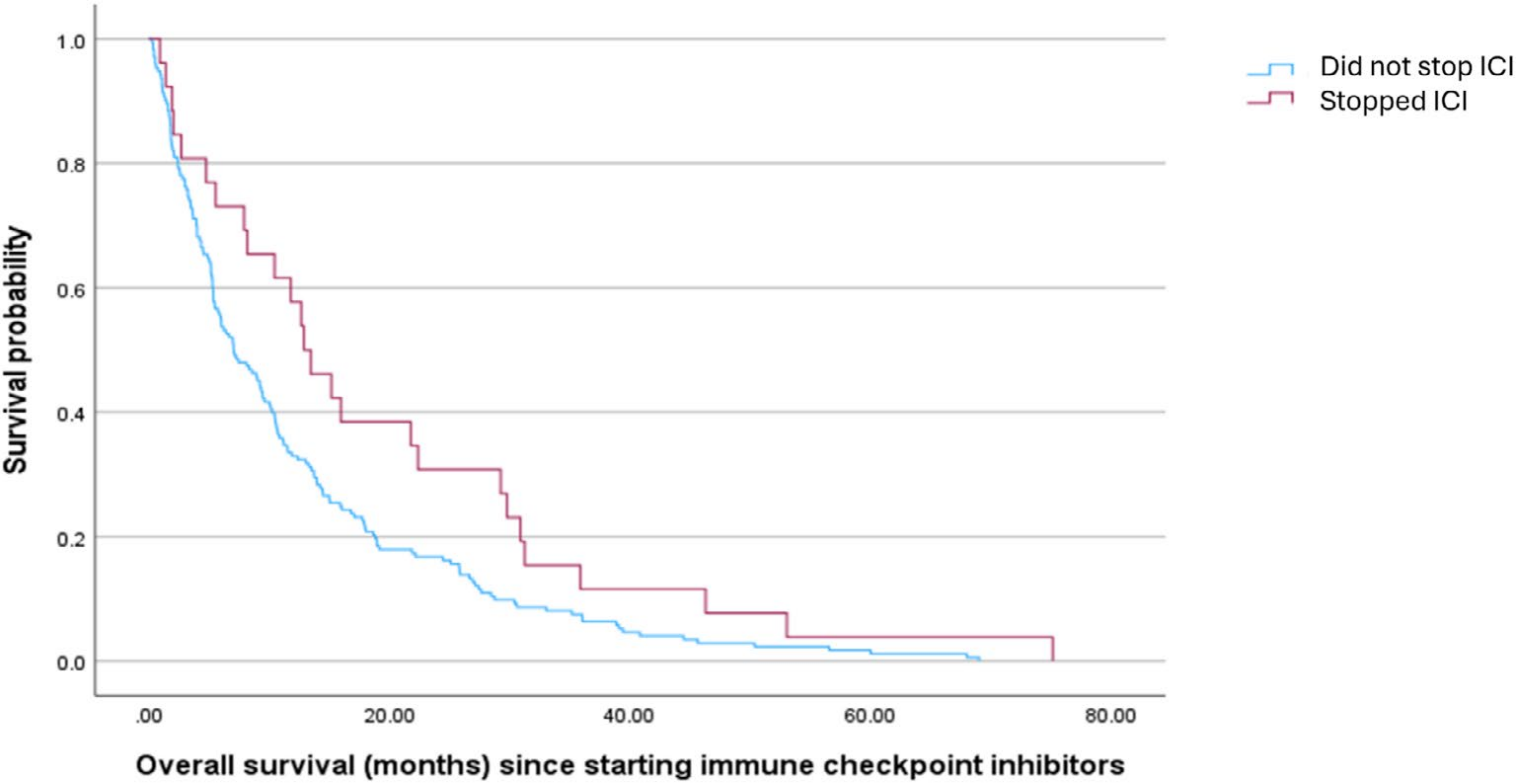
Overall survival (OS) with immune checkpoint inhibitors depending on the incidence of immune‐related toxicity and stopping therapy in 344 melanoma patients who received immune checkpoint inhibitors (ICI) for unresectable or advanced disease in University Hospitals Sussex NHS Foundation Trust (UHSussex). The median OS was significantly longer for patients who experienced immune‐related toxicity and their immune checkpoint inhibitors therapy was stopped, with a mean difference of 12.2 months in patients who did not experience immune‐related toxicity and stopping therapy compared to 19.4 months for patients experienced immune‐related toxicity and stopping therapy (*p* = 0.027).

**FIGURE 6:**
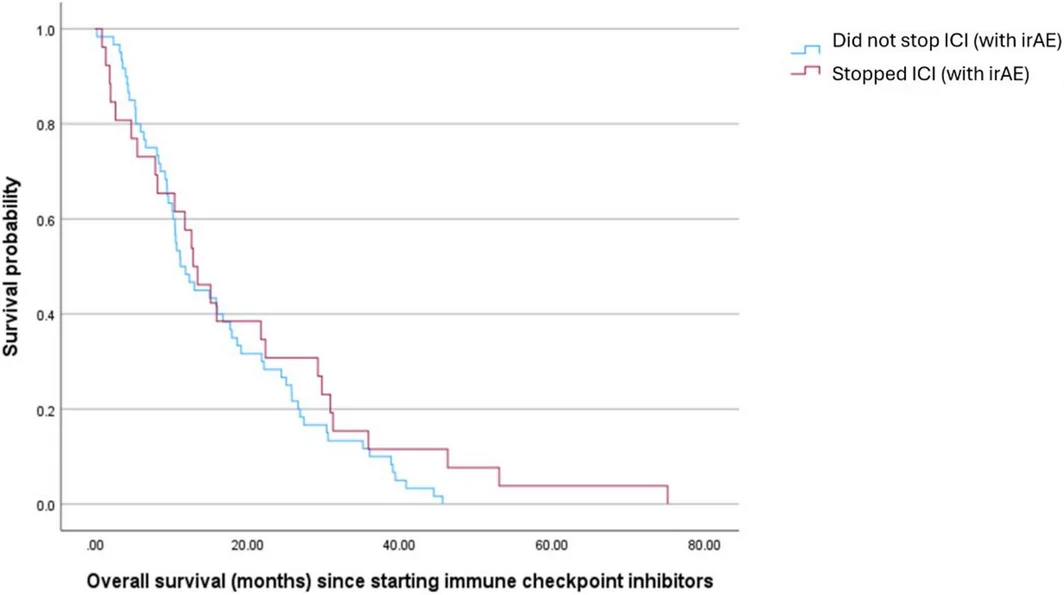
Overall survival (OS) with immune checkpoint inhibitors depending on the incidence of immune‐related toxicity and stopping therapy in patients who developed immune‐related adverse events (irAEs) in 184 melanoma patients who experience IrAEs when received immune checkpoint inhibitors (ICI) for unresectable or advanced disease in University Hospitals Sussex NHS Foundation Trust (UHSussex). The median OS was not significantly different (*p* = 0.285) with mean survival = 19.4 months for patients who developed immune‐related toxicity and requiring ICI stop vs. 16.6 months for patients who developed irAEs without immune‐related toxicity and not requiring ICI stop.

We apologize for this error.

