# Supplementary material for: Immune‐Related Adverse Events and Therapeutic Outcomes After Stopping Immune Checkpoint Inhibitors due to Toxicity Among Patients With Metastatic Melanoma (University Hospitals Sussex)
Source: Cancer Med. 2026 Jul 22;15(7):e72119. doi: 10.1002/cam4.72119 (PMC13389604; doi:10.1002/cam4.72119)
Supplement: Supplementary file 1 — Table S1: Summary of grade distribution, organ‐specific toxicities, steroid requirement, hospitalisation and discontinuation rates in 344 metastatic melanoma patients received ICI for unresectable or advanced disease in University Hospitals Sussex NHS Foundation Trust (UHSussex) from the period between October 2011 and December 2022. [file CAM4-15-e72119-s001.docx]

Supplementary Table 1 Summary of grade distribution, organ-specific toxicities, steroid requirement, hospitalization, and discontinuation rates in 344 metastatic melanoma patients received ICI for unresectable or advanced disease in University Hospitals Sussex NHS Foundation Trust (UHSussex) from the period between October 2011 and December 2022

| Disease site | Stage | Outcome of ICI | IrAE 1 | IrAE 1 Grade | IrAE 2 | IrAE 2 Grade | IrAE 3 | IrAE 3 Grade | Corticosteroid Use | Infliximab required? |
| --- | --- | --- | --- | --- | --- | --- | --- | --- | --- | --- |
| Nasal mucosa | 4 | Progression | None |  |  |  |  |  | NA | NA |
| Left arm | 4 | Completion of planned therapy | Endocrine |  |  |  |  |  | NA | NA |
| Ocular | 4 | Progression |  |  |  |  |  |  | NA | NA |
| Left abdomen | 4 | Progression |  |  |  |  |  |  | NA | NA |
| Lower back | 4 | Therapy stopped due to toxicity | GIT | 3 | Skin | 2 |  |  | PO Prednisolone FLAT dose | No |
| Right breast | 4 | Progression | GIT | 4 |  |  |  |  | IV Methylprednisolone 500mg | No |
| Forearm | 3c | Progression |  |  |  |  |  |  | NA | NA |
| Deltoid | 4 | Progression |  |  |  |  |  |  | NA | NA |
| Right back | 4 | Therapy stopped due to toxicity | Other | 4 |  |  |  |  | NA | NA |
| Abdomen | 4 | Progression | Skin | 2 |  |  |  |  | NA | NA |
| Left thigh | 4 | Completion of planned therapy | |  |  |  |  |  | NA | NA |
| Unidentified | 4 | Completion of planned therapy | |  |  |  |  |  | NA | NA |
| Unidentified | 4 | Completion of planned therapy | |  |  |  |  |  | NA | NA |
| Ear ((brain, liver, lung, soft tissue) | 4 | Progression |  |  |  |  |  |  | NA | NA |
| Right ankle | 4 | Therapy stopped due to toxicity | GIT | 3 | Endocrine |  |  |  | IV Methylprednisolone 500mg | No |
| Brain met. | 4 | Death |  |  |  |  | None |  | NA | NA |
| Nasal mucosa | 4 | Progression | Skin | 2 |  |  |  |  | NA | NA |
| Right shin | 3 | Therapy stopped due to toxicity | Hepatic | 2 | Renal | 3 |  |  | NA | NA |
| Ocular | 4 | Progression | Skin | 2 |  |  |  |  | NA | NA |
| Multiple pulmonary, liver, peritoneal lymph node and subcutaneous metastases, brain met. | 4 | Therapy stopped due to toxicity | GIT | 3 |  |  |  |  | IV Methylprednisolone 500mg | No |
| Left arm | 4 | Completion of planned therapy | |  |  |  |  |  | NA | NA |
| Hairline | 4 | Progression |  |  |  |  |  |  | NA | NA |
| No data | 4 | Progression |  |  |  |  | None |  | NA | NA |
| Scalp | 4 | Death |  |  |  |  |  |  | NA | NA |
| Right elbow | 4 | Progression | Hepatic | 2 |  |  |  |  | NA | NA |
| Right shoulder | 4 | Progression |  |  |  |  |  |  | NA | NA |
| Multiple nodal disease and liver metastasis No brain metastasis | 4 | Death |  |  |  |  | None |  | NA | NA |
| Left cheek | 4 | Progression |  |  |  |  |  |  | NA | NA |
| No data | 4 | Progression |  |  |  |  | None |  | NA | NA |
| Primary not found | 4 | Completion of planned therapy | GIT | 3 | Skin | 1 |  |  | PO Prednisolone FLAT dose | No |
| Back | 4 | Progression |  |  |  |  |  |  | NA | NA |
| Left temple | 4 | Completion of planned therapy | |  |  |  |  |  | NA | NA |
| Left cheek | 4 | Hospitalisation |  |  |  |  |  |  | NA | NA |
| Left arm | 3 | Progression |  |  |  |  |  |  | NA | NA |
| Scalp | 3 | Completion of planned therapy | |  |  |  |  |  | NA | NA |
| Left neck | 4 | Therapy stopped due to toxicity | GIT | 3 |  |  |  |  | PO Prednisolone FLAT dose | No |
| Left shoulder | 4 | Completion of planned therapy | |  |  |  |  |  | NA | NA |
| Not stated | 4 | Progression | GIT | 3 |  |  |  |  | IV Methylprednisolone 1-2mg/kg/day | No |
| Recurrent metastatic malignant melanoma left neck | 4 | Therapy stopped due to toxicity | GIT | 3 |  |  |  |  | IV Methylprednisolone 1-2mg/kg/day | Yes |
| Right neck | 4 | Death |  |  |  |  |  |  | NA | NA |
| Anus | 4 | Hospitalisation | Skin | 1 |  |  |  |  | NA | NA |
| Left arm | 4 | Completion of planned therapy | Endocrine | 2 |  |  |  |  | NA | NA |
| Ocular | 4 | Therapy stopped due to toxicity | GIT | 3 | GIT | 1 |  |  | IV Methylprednisolone 500mg | No |
| Left leg | 4 | Death |  |  |  |  |  |  | NA | NA |
| Lower back | 3d | Completion of planned therapy | Endocrine | 2 |  |  |  |  | NA | NA |
| Scalp | 4 | Completion of planned therapy | |  |  |  |  |  | NA | NA |
| Right calf | 4 | Therapy stopped due to toxicity | Endocrine | 3 |  |  |  |  | NA | NA |
| Lung metastasis, new liver metastasis, adrenal metastasis and right subcutaneous deposit in the buttock | 4 | Hospitalisation |  |  |  |  | None |  | NA | NA |
| Not stated | 4 | Therapy stopped due to toxicity | Renal |  |  |  |  |  | NA | NA |
| Extensive metastatic disease with disease in right axilla, subcutaneous disease, neck nodes, possible liver metastasis, possible lung metastasis, nodal disease below the diaphragm, and also tiny peritoneal nodules consistent with metastasis | 4 | Death |  |  |  |  | None |  | NA | NA |
| Ocular | 4 | Therapy stopped due to toxicity | Hepatic | 1 |  |  |  |  | NA | NA |
| Left neck | 4 | Therapy stopped due to toxicity | Neuro. | 3 |  |  |  |  | NA | NA |
| Multiple new sites of melanoma deposits in leg | 4 |  | MSK | 1 |  |  |  |  | NA | NA |
| Mole- not stated | 4 | Therapy stopped due to toxicity | GIT |  | MSK |  |  |  | IV Methylprednisolone 500mg | Yes |
| Ear | 4 | Completion of planned therapy | Hepatic | 2 | Endocrine | 2 |  |  | NA | NA |
| No data | 4 | Completion of planned therapy | |  |  |  | None |  | NA | NA |
| Lung met | 4 | Therapy stopped due to toxicity | MSK | 2 | GIT | 1 |  |  | PO Prednisolone FLAT dose | No |
| Left chest | 4 | Completion of planned therapy | GIT | 1 |  |  |  |  | PO Prednisolone FLAT dose | NA |
| Left chest | 2 | Progression | Endocrine | 2 |  |  |  |  | NA | NA |
| Left armpit | 4 | Completion of planned therapy | MSK | 2 |  |  |  |  | NA | NA |
| Left arm | 4 | Completion of planned therapy | GIT | 1 |  |  |  |  | PO Prednisolone FLAT dose | No |
| Abdomen | 4 | Progression |  |  |  |  |  |  | NA | NA |
| Solitary left space occupying lesion (brain met) | 4 | Progression |  |  |  |  |  |  | NA | NA |
| Not stated | 4 | Progression | Hepatic | 3 | Respiratory |  |  |  | NA | NA |
| Not stated | 4 | Progression | Endocrine |  |  |  |  |  | NA | NA |
| Abdomen | 4 | Therapy stopped due to toxicity | GIT |  |  |  |  |  | IV Methylprednisolone 500mg | No |
| Not stated | 4 | Therapy stopped due to toxicity | Hepatic |  |  |  |  |  | NA | NA |
| Forehead | 4 | Completion of planned therapy | |  |  |  |  |  | NA | NA |
| Abdomen | 4 | Progression |  |  |  |  |  |  | NA | NA |
| Left foot | 4 | Progression |  |  |  |  |  |  | NA | NA |
| Back | 4 | Completion of planned therapy | Endocrine | 2 |  |  |  |  | NA | NA |
|  | 4 | Completion of planned therapy | Skin | 1 |  |  |  |  | NA | NA |
| Right knee | 4 | Completion of planned therapy | |  |  |  |  |  | NA | NA |
| Nasal tip | 4 | Completion of planned therapy | |  |  |  |  |  | NA | NA |
| Scalp | 4 | Progression |  |  |  |  |  |  | NA | NA |
| Right buttock | 4 | Therapy stopped due to toxicity | GIT | 3 | Hepatic | 2 |  |  | IV Methylprednisolone 1-2mg/kg/day | No |
| Back | 4 | Therapy stopped due to toxicity | GIT | 3 | Hepatic | 2 |  |  | IV Methylprednisolone 500mg | No |
| Recurrent melanoma right back | 4 | Therapy stopped due to toxicity | Skin | 1 | MSK | 2 |  |  | NA | NA |
| Oesophagus | 4 | Progression |  |  |  |  |  |  | NA | NA |
| Abdomen | 4 | Progression |  |  |  |  |  |  | NA | NA |
| Chest | 4 | Completion of planned therapy | |  |  |  |  |  | NA | NA |
| Left foot | 4 | Progression | Endocrine | 2 |  |  |  |  | NA | NA |
| Right thigh | 4 | Progression | GIT | 2 |  |  |  |  | NA | NA |
| Right cheek | 4 | Therapy stopped due to toxicity | GIT | 1 | Hepatic | 1 |  |  | NA | NA |
| Left heel | 4 | Completion of planned therapy | |  |  |  |  |  | NA | NA |
| Right thigh | 4 | Progression | MSK | 3 | Skin | 2 |  |  | NA | NA |
| Neck | 4 | Progression |  |  |  |  | None |  | NA | NA |
| Nasal mucosa | 4 | Progression |  |  |  |  |  |  | NA | NA |
| Ocular | 4 | Progression |  |  |  |  |  |  | NA | NA |
| Right forearm | 4 | Progression |  |  |  |  |  |  | NA | NA |
| Right shoulder | 4 | Completion of planned therapy | Endocrine | 2 |  |  |  |  | NA | NA |
| Back | 4 | Death |  |  |  |  |  |  | NA | NA |
| Right thigh | 4 | Death |  |  |  |  |  |  | NA | NA |
| Back | 4 | Progression | GIT | 3 |  |  |  |  | IV Methylprednisolone 500mg | No |
| Left arm | 4 | Therapy stopped due to toxicity | MSK |  |  |  |  |  | NA | NA |
| Right cheek | 4 | Progression | MSK |  |  |  |  |  | NA | NA |
| Abdomen | 4 | Therapy stopped due to toxicity | GIT | 2 | Hepatic | 4 |  |  | IV Methylprednisolone 1-2mg/kg/day | No |
| Neck | 4 | Progression | GIT | 2 |  |  |  |  | PO Prednisolone FLAT dose | No |
| Left axillary sentinel node | 4 | Death |  |  |  |  |  |  | NA | NA |
| Lung | 4 | Therapy stopped due to toxicity | GIT | 3 |  |  |  |  | IV Methylprednisolone 500mg | Yes |
| Left buttock | 4 | Progression |  |  |  |  |  |  | NA | NA |
| Ocular | 4 | Progression |  |  |  |  |  |  | NA | NA |
| Abdomen | 4 | Death |  |  |  | NA | None |  | NA | NA |
| Left leg | 4 | Completion of planned therapy | |  |  |  |  |  | NA | NA |
| Chest | 3 | Hospitalisation |  |  |  |  |  |  | NA | NA |
| Left heel | 4 | Completion of planned therapy | |  |  |  |  |  | NA | NA |
| Abdomen | 4 | Therapy stopped due to toxicity | Hepatic | 2 |  |  |  |  | NA | NA |
| Extensive liver metastases, peritoneal disease, ascites and pleural fluid, brain met. | 4 | Death |  |  |  |  | None |  | NA | NA |
| Breast | 4 | Progression |  |  |  |  |  |  | NA | NA |
| Liver | 4 | Therapy stopped due to toxicity | GIT | 3 |  |  |  |  | NA | NA |
| Multiple brain metastasis and multiple subcutaneous deposits, liver metastasis, splenic metastasis, peritoneal disease, mediastinal nodes and small lung metastasis | 4 | Death |  |  |  |  | None |  | NA | NA |
| Left temple | 4 | Progression |  |  |  |  |  |  | NA | NA |
| Brain, bones, spleen, and paraaortic lymph nodes. | 4 | Completion of planned therapy | GIT | 1 | Other | NA |  |  | PO Prednisolone FLAT dose | NA |
| Mutated malignant melanoma right shoulder | 4 | Therapy stopped due to toxicity | Other | NA |  |  |  |  | NA | NA |
| Right forearm | 4 | Progression | GIT | NA |  |  |  |  | NA | No |
| Lung & brain met. (central back oct 2016) | 4 | Death |  |  |  |  | None |  | NA | NA |
| Malignant melanoma with a new brain liver, lung metastases. | 3 | Progression |  |  |  |  |  |  | NA | NA |
| Liver met. (mid back Feb. 21) | 4 | Hospitalisation |  |  |  |  |  |  | NA | NA |
| Back | 4 | Progression |  |  |  |  |  |  | NA | NA |
| Malignant melanoma excised anterior abdominal wall RMH. | 4 | Completion of planned therapy | no toxicities |  |  |  |  |  | NA | NA |
| CT -progression with symptomatic brain met. (2006 right flank) | 4 | Therapy stopped due to toxicity | GIT | 3 |  |  |  |  | IV Methylprednisolone 1-2mg/kg/day | Yes |
| Left posterior chest wall. | 4 | Therapy stopped due to toxicity | GIT | 2 |  |  |  |  | PO Prednisolone FLAT dose | No |
| Right axillary node | 4 | Therapy stopped due to toxicity | GIT | 3 |  |  |  |  | IV Methylprednisolone 1-2mg/kg/day | NA |
| Extensive intra and extra cranial | 4 | Therapy stopped due to toxicity | |  |  |  |  |  | IV Methylprednisolone 1-2mg/kg/day | NA |
| Right calf | 4 | Therapy stopped due to toxicity | Endocrine |  |  |  |  |  | NA | NA |
| Liver | 4 | Progression |  |  |  |  |  |  | PO Prednisolone FLAT dose | NA |
| Left axillary sentinel node | 4 | Therapy stopped due to toxicity | Other |  | MSK | 2 |  |  | PO Prednisolone FLAT dose | NA |
| Left ankle with skin graft | 4 | Progression |  |  |  |  |  |  | PO Prednisolone FLAT dose | NA |
| Left eyelid | 4 | Completion of planned therapy | Cardiac |  |  |  |  |  | NA | NA |
| Left foot | 4 | Hospitalisation | GIT |  |  |  |  |  | NA | NA |
| Multiple bilateral brain metastasis, lung metastasis, peritoneal disease, right atrial cardiac metastasis and para hilar/mediastinal lymphadenopathy. | 4 | Progression | GIT | 2 | Hepatic | 2 |  |  | PO Prednisolone FLAT dose | No |
| Eft and right sided neck nodes | 3 | Therapy stopped due to toxicity | Cardiac |  |  |  |  |  | NA | NA |
| Right cheek | 4 | Therapy stopped due to toxicity | Endocrine |  |  |  |  |  | PO Prednisolone FLAT dose | NA |
| Brain metastases. | 4 |  |  |  |  |  |  |  | PO Prednisolone FLAT dose | NA |
| Right sided lung | 4 | Progression |  |  |  |  |  |  | NA | NA |
| Right leg | 4 | Progression | Endocrine |  |  |  |  |  | NA | NA |
| Brain metastasis | 4 | Progression |  |  |  |  |  |  | NA | NA |
| Cerebellar, lung, adrenal, lymph nodes and soft tissue metastasis | 4 | Progression | GIT |  |  |  |  |  | PO Prednisolone FLAT dose | NA |
| Nasal tumour | 4 | Progression |  |  |  |  |  |  | NA | NA |
| Left anterior superior chest wall | 4 | Therapy stopped due to toxicity | GIT | 2 | GIT | 3 |  |  | NA | NA |
| Distal right thigh | 4 | Progression |  |  |  |  |  |  | Other | NA |
| Multiple brain metastases. | 4 | Progression |  |  |  |  |  |  | NA | NA |
| Right groin node | 4 | Therapy stopped due to toxicity | MSK | 2 | Skin | 1 |  |  | PO Prednisolone FLAT dose | NA |
| Liver met. | 4 | Therapy stopped due to toxicity | Skin | 1 | Respiratory | 3 |  |  | NA | NA |
| Left leg | 4 | Therapy stopped due to toxicity | GIT | 2 |  |  |  |  | NA | NA |
| Brain | 4 | Therapy stopped due to toxicity | Respiratory |  |  |  |  |  | NA | NA |
| G multiple liver metastasis, multiple lung metastasis, mediastinal node and also multiple subcutaneous deposits | 4 | Progression |  |  |  |  |  |  | NA | NA |
| Excised back | 4 | Progression |  |  |  |  |  |  | IV Methylprednisolone 500mg | Yes |
| Left shoulder | 4 | Progression |  |  |  |  |  |  | NA | NA |
| Excised chin. | 4 | Therapy stopped due to toxicity | GIT |  |  |  |  |  | IV Methylprednisolone 500mg | Yes |
| Trunk, Dec 2021 lung met. | 4 | Progression |  |  |  |  | None |  | NA | NA |
| March 2017 multiple liver metastasis, small volume lung metastasis after excision right heel and right groin Jan 2017 | 4 | Death |  |  |  |  | None |  | NA | NA |
| Resected from the back | 4 | Death | Respiratory |  |  |  |  |  | Other | NA |
| C choroidal melanoma | 4 | Progression | Skin |  |  |  |  |  | PO Prednisolone FLAT dose | NA |
| Right calf | 4 | Progression |  |  |  |  |  |  | PO Prednisolone FLAT dose | NA |
| Left back | 4 | Completion of planned therapy | |  |  |  |  |  | NA | NA |
| Brain and right axilla | 4 | Progression |  |  |  |  | None |  | NA | NA |
| Skin | 4 | Progression |  |  |  |  |  |  | NA | NA |
| Left leg | 4 | Progression |  |  |  |  |  |  | NA | NA |
| Right posterior calf | 4 | Progression |  |  |  |  |  |  | NA | NA |
| Left sided cervical lymphadenopathy | 4 | Progression |  |  |  |  |  |  | NA | NA |
| Skin | 4 | Progression | Hepatic | 3 |  |  |  |  | NA | NA |
| Back, liver, brain | 4 | Death |  |  |  |  |  |  | NA | NA |
| Right thumb | 4 | Progression |  |  |  |  |  |  | IV Methylprednisolone 1-2mg/kg/day | No |
| Left forearm | 4 | Progression | GIT |  |  |  |  |  | PO Prednisolone FLAT dose | NA |
| Brain | 4 | Progression |  |  |  |  |  |  | Other | NA |
| Right posterior shoulder. | 4 | Progression |  |  |  |  |  |  | Other | NA |
| Liver & renal met | 4 | Progression |  |  |  |  |  |  | NA | NA |
| Right arm | 4 | Death |  |  |  |  |  |  | Other | NA |
| Left upper back | 4 | Therapy stopped due to toxicity | GIT | 3 |  |  |  |  | IV Methylprednisolone 1-2mg/kg/day | Yes |
| Right back | 4b | Progression |  |  |  |  |  |  | Other | NA |
| Left leg | 3 | Therapy stopped due to toxicity | |  |  |  |  |  | NA | NA |
| Left neck/shoulder | 3b | Therapy stopped due to toxicity | Skin | 2 | Hepatic | 3 |  |  | IV Methylprednisolone 500mg | NA |
| Left anterior abdominal wall. | 3b | Progression |  |  |  |  |  |  | Other | NA |
| Right shoulder | 4b | Progression |  |  |  |  |  |  | NA | NA |
| Right scalp | 4a | Progression |  |  |  |  |  |  | NA | NA |
| Scalp melanoma | 4b | Completion of planned therapy | GIT |  |  |  |  |  | Other | NA |
| Scalp | 4b | Progression |  |  |  |  |  |  | Other | NA |
| Upper arm | 4b | Progression |  |  |  |  |  |  | NA | NA |
| Left arm | 4 | Completion of planned therapy | |  |  |  |  |  | NA | NA |
| Central upper posterior trunk | 4 | Therapy stopped due to toxicity | Hepatic | 3 |  |  |  |  | IV Methylprednisolone 500mg | NA |
| Right shoulder | 3 | Therapy stopped due to toxicity | Respiratory | 3 |  |  |  |  | NA | NA |
| Left ear | 4 | Therapy stopped due to toxicity | Hepatic | 3 |  |  |  |  | IV Methylprednisolone 1-2mg/kg/day | NA |
| Recurrence left upper arm - imaging- metastatic disease with 2 small new lung metastases and small splenic metastases | 4 | Therapy stopped due to toxicity | GIT | 2 | Endocrine | 2 |  |  | IV Methylprednisolone 1-2mg/kg/day | No |
| Left femur and knee | 4 | Progression | Skin |  |  |  |  |  | NA | NA |
| Brain | 4 | Therapy stopped due to toxicity | GIT | 3 | Hepatic | 2 |  |  | IV Methylprednisolone 1-2mg/kg/day | Yes |
| Back | 4 | Therapy stopped due to toxicity | Other | 3 |  |  |  |  | NA | NA |
| Right axilla | 4 | Progression |  |  |  |  | None |  | NA | NA |
| Left thigh | 4 | Progression | GIT | 2 |  |  |  |  | NA | NA |
| Progression on dabrafenib (oct 2017) (diagnosis 2011) | 4 | Progression | Skin | 2 | Endocrine | 2 |  |  | NA | NA |
| Brain, liver, lung met. (2011-right forehead) | 4 | Progression |  |  |  |  | None |  | NA | NA |
| Lung (Jan 2014-right eye proptosis) | 4 | Progression | Hepatic | 3 | Endocrine | 2 | None |  | NA | NA |
| Inoperable left groin | 3c | Therapy stopped due to toxicity | GIT | 2 |  |  |  |  | PO Prednisolone 0.5-1mg/kg/day | NA |
| Left groin, 2017 dabrafenib, recurrence May 2018 | 4 | Progression |  |  |  |  | None |  | NA | NA |
| Abdominal wall, right axilla, Aug 2016 recurrence + liver met. | 4 | Completion of planned therapy | |  |  |  | None |  | NA | NA |
| Lung met. | 4 | Progression | Skin | 1 |  |  |  |  | NA | NA |
| Vaginal wall (2007 excised anterior chest wall) | 4 | Progression |  |  |  |  | None |  | NA | NA |
| Recurrent right groin (2015 lower back) | 4 | Completion of planned therapy | |  |  |  | None |  | NA | NA |
| Lung met | 4 | Completion of planned therapy | Skin | 1 |  |  |  |  | NA | NA |
| Recurrence (Feb. 2017 excised right leg melanoma) | 3c | Completion of planned therapy | Endocrine | 2 | Hepatic | 2 |  |  | NA | NA |
| Right shoulder (Nov 2020 lung met) | 4 | Completion of planned therapy | |  |  |  | None |  | NA | NA |
| Left scalp | 3 | Completion of planned therapy | |  |  |  | None |  | NA | NA |
| Forehead (recurrence parotid) | 3 | Progression | Endocrine | 2 |  |  |  |  | NA | NA |
| Lung met. | 4 | Progression |  |  |  |  | None |  | NA | NA |
| Brain met | 4 | Progression | Hepatic | 2 |  |  |  |  | NA | NA |
| Lung met. & spinal cord compression | 4 | Progression | Hepatic | 3 | GIT | 1 |  |  | IV Methylprednisolone 1-2mg/kg/day | No |
| Lung nodule (April 2017 -right ear) | 4 | Therapy stopped due to toxicity | GIT | 3 | Hepatic | 2 |  |  | IV Methylprednisolone 1-2mg/kg/day | Yes |
| Lung met, bilateral axillary lymph nodes | 4 | Progression | Hepatic | 2 |  |  |  |  | NA | NA |
| Liver & spleen met. (sept 2018 right abdomen) | 4 | Progression |  |  |  |  | None |  | NA | NA |
| Liver met. (choroidal melanoma April 2014) | 4 | Progression |  |  |  |  | None |  | NA | NA |
| Multiple lungs met., brain met. | 4 | Progression |  |  |  |  | None |  | NA | NA |
| Buccal mucosa | 4 | Progression |  |  |  |  | None |  | NA | NA |
| Pelvic mass, liver met | 4 | Progression |  |  |  |  | None |  | NA | NA |
| Mets | 4 | Therapy stopped due to toxicity | MSK | 3 |  |  |  |  | NA | NA |
| Scalp. Dec 2022 lung met. | 4 | Therapy stopped due to toxicity | MSK | 2 | Cardiac | 2 |  |  | NA | NA |
| Left parotid lymph node, multiple bone met. (left ear sept 2019) | 4 | Progression | Hepatic | 1 | GIT | 2 |  |  | PO Prednisolone FLAT dose | NA |
| Forehead, left orbit |  | Progression | Skin | 1 |  |  |  |  | NA | NA |
| Right neck | 3 | Hospitalisation |  |  |  |  | None |  | NA | NA |
| Axillary recurrence | 4 | Progression |  |  |  |  | None |  | NA | NA |
| Brain met | 4 | Progression |  |  |  |  | None |  | NA | NA |
| Liver & lung met. | 4 | Progression |  |  |  |  | None |  | NA | NA |
| Left flank | 4 | Progression | MSK | 2 |  |  |  |  | NA | NA |
| Liver, lung axillary met. | 4 | Therapy stopped due to toxicity | Hepatic | 3 |  |  |  |  | NA | NA |
| Chest wall | 4 | Therapy stopped due to toxicity | Respiratory | 4 | Endocrine | 2 |  |  | NA | NA |
| Right groin | 4 | Hospitalisation | Renal | 2 |  |  |  |  | NA | NA |
| Lung met. | 4 | Death | Endocrine | 2 |  |  |  |  | NA | NA |
| Neck, lungs, spine | 4 | Therapy stopped due to toxicity | GIT | 3 |  |  |  |  | PO Prednisolone FLAT dose | Yes |
| Cheek | 4 | Therapy stopped due to toxicity | Hepatic | 2 |  |  |  |  | NA | NA |
| Liver, lung, brain and bone metastases. | 4 | Therapy stopped due to toxicity | GIT | 3 |  |  |  |  | PO Prednisolone FLAT dose | NA |
| Lung met. | 4 | Completion of planned therapy | |  |  |  | None |  | NA | NA |
| Lung met. | 4 | Therapy stopped due to toxicity | Skin | 1 | Neuro. | 2 |  |  | NA | NA |
| Multiple brain metastases, hepatic, bone and soft tissue subcutaneous and intramuscular deposits | 4 | Death |  |  |  |  | None |  | NA | NA |
| Groin (2007 - thigh) | 4 | Completion of planned therapy | GIT | 2 | Endocrine | 2 |  |  | NA | NA |
| Back, Aug 2020 lung met. | 4 | Progression |  |  |  |  | None |  | NA | NA |
| Lung met. (2015 temple) | 4 | Completion of planned therapy | Skin | 1 |  |  |  |  | NA | NA |
| Neck (inoperable) | 4 | Progression | GIT | 2 |  |  |  |  | PO Prednisolone FLAT dose | NA |
| Brain met., lung met. | 4 | Progression |  |  |  |  |  |  | NA | NA |
| Neck | 3 | Completion of planned therapy | |  |  |  | None |  | NA | NA |
| Upper thigh | 3 | Therapy stopped due to toxicity | GIT | 3 | Endocrine | 2 |  |  | IV Methylprednisolone 1-2mg/kg/day | Yes |
| Spinal & bone met., lung, adrenal met, no brain met. (2008 melanoma posterior neck) | 4 | Progression | GIT | 2 |  |  |  |  | PO Prednisolone FLAT dose | NA |
| Right shoulder | 3 | Therapy stopped due to toxicity | Hepatic | 3 | GIT | 2 |  |  | IV Methylprednisolone 1-2mg/kg/day | NA |
| Multiple met. Face, trunk, limbs | 4 | Therapy stopped due to toxicity | GIT | 2 |  |  |  |  | PO Prednisolone FLAT dose | NA |
| Right leg, recurrence Sep 2016 excised, May 2017, right iliac lymph node | 3 | Progression | GIT | 2 | Endocrine | 2 | Hepatic | 2 | PO Prednisolone FLAT dose | NA |
| Right axillary lymph node (2015 right arm) | 4 | Completion of planned therapy | Hepatic | 3 |  |  |  |  | NA | NA |
| Progression on BRAF/MEK inhibitors (April 2018 to Aug 2020) | 4 | Progression |  |  |  |  | None |  | NA | NA |
| Lung, brain & peritoneal met. | 4 | Progression |  |  |  |  |  |  | NA | NA |
| Right axillary node (Nov 2014 back) | 4 | Therapy stopped due to toxicity | GIT | 2 |  |  |  |  | PO Prednisolone FLAT dose | NA |
| Liver met. |  | Progression | GIT | 3 |  |  |  |  | PO Prednisolone FLAT dose | No |
| Liver met., on surveillance for choroidal melanoma right (June 2018) | 4 | Progression |  |  |  |  | None |  | NA | NA |
| Unidentified primary, brain met. | 4 | Progression |  |  |  |  | None |  | NA | NA |
| Mediastinal lymphadenopathy and subcutaneous deposits, Nov 2018 solitary brain met | 4 | Completion of planned therapy | |  |  |  | None |  | NA | NA |
| Progression of groin, new lung met | 3 | Progression |  |  |  |  | None |  | NA | NA |
| Chest wall | 4 | Death |  |  |  |  | None |  | NA | NA |
| Liver & bone met, ocular melanoma 2015 |  | Progression | MSK | 2 | Hepatic | 2 |  |  | NA | NA |
| Axilla, subpectoral lymph adenopathy, arm | 3 | Completion of planned therapy | |  |  |  | None |  | NA | NA |
| Axilla recurrence + lung met, 2012 shoulder | 4 | Progression |  |  |  |  | None |  | NA | NA |
| Left arm, recurrence axilla Oct 21, Jan 22 brain & liver met. | 4 | Progression |  |  |  |  | None |  | NA | NA |
| Nasal polyp | 4 | Therapy stopped due to toxicity | Other | 3 |  |  |  |  | NA | NA |
| Brain met | 4 | Death |  |  |  |  | None |  | NA | NA |
| Axilla | 4 | Progression | Endocrine | 2 |  |  |  |  | NA | NA |
| Lung met. | 4 | Therapy stopped due to toxicity | Skin | 1 | Endocrine | 2 |  |  | NA | NA |
| Rectum, Feb. 2015 - inguinal node (started immunotherapy) | 4 | Progression | Endocrine | 2 |  |  |  |  | NA | NA |
| Scalp | 4 | Completion of planned therapy | |  |  |  | None |  | NA | NA |
| Neck, kidney (unidentified primary site) | 4 | Progression | Skin | 1 |  |  |  |  | NA | NA |
| Liver met | 4 | Progression |  |  |  |  | None |  | NA | NA |
| Brain - space occupying lesion, Unknown primary | 4 | Progression |  |  |  |  | None |  | NA | NA |
| Left ear | 4 | Completion of planned therapy | MSK | 2 |  |  |  |  | NA | NA |
| Left elbow | 4 | Death |  |  |  |  | None |  | NA | NA |
| Groin node (April 2011) | 4 | Progression |  |  |  |  | None |  | NA | NA |
| Unknown primary | 4 | Therapy stopped due to toxicity | Skin | 1 | GIT | 3 |  |  | IV Methylprednisolone 1-2mg/kg/day | NA |
| Lung met. (2014 right back) | 4 | Progression |  |  |  |  | None |  | NA | NA |
| Recurrence left calf (2006 excised left calf) | 3 | Progression | Hepatic | 2 | Endocrine | 2 |  |  | NA | NA |
| Left heel | 4 | Progression |  |  |  |  | None |  | NA | NA |
| Left upper back | 4 | Progression | GIT | 2 | Skin | 2 |  |  | PO Prednisolone 0.5-1mg/kg/day | NA |
| Right groin node (April 2013 right lower leg) | 4 | Progression |  |  |  |  | None |  | NA | NA |
| Lung nodule (2012 left foot) | 4 | Progression | GIT | 2 |  |  |  |  | PO Prednisolone 0.5-1mg/kg/day | NA |
| Sept 2019 - presumed triple negative breast cancer (Oct 2021- headache- brain & lung met) | 4 | Progression | GIT | 2 |  |  |  |  | PO Prednisolone 0.5-1mg/kg/day | NA |
| Left calf | 4 | Completion of planned therapy | |  |  |  | None |  | NA | NA |
| Met (Aug 2012 back) | 4 | Therapy stopped due to toxicity | GIT | 1 |  |  |  |  | NA | NA |
| Left back | 4 | Progression |  |  |  |  | None |  | NA | NA |
| Met (2007 - right cheek) | 4 | Therapy stopped due to toxicity | Endocrine | 2 | GIT | 1 |  |  | NA | NA |
| Right big toe | 4 | Progression | GIT | 2 |  |  |  |  | IV Methylprednisolone 1-2mg/kg/day | NA |
| Left flank | 4 | Progression |  |  |  |  | None |  | NA | NA |
| Left inguinal resection | 4 | Therapy stopped due to toxicity | Endocrine | 4 |  |  |  |  | NA | NA |
| Left groin node | 4 | Progression |  |  |  |  | None |  | NA | NA |
| Right upper arm | 4 | Progression |  |  |  |  | None |  | NA | NA |
| Abdominal wall | 4 | Progression | Skin | 1 |  |  |  |  | NA | NA |
| Left calf March 2018, then left groin Oct – met. | 3 | Progression | Neuro. | 3 | Neuro. | 1 |  |  | NA | NA |
| Liver, lung, stomach | 4 | Progression | GIT | 1 | Hepatic | 2 |  |  | PO Prednisolone 0.5-1mg/kg/day | NA |
| Lung nodule (2012 left foot) | 4 | Progression | GIT | 1 |  |  |  |  | NA | NA |
| Liver & lung met. (dec 20122 upper back) | 4 | Completion of planned therapy | |  |  |  | None |  | NA | NA |
| Left calf | 3 | Progression |  |  |  |  | None |  | NA | NA |
| Left thigh | 4 | Completion of planned therapy | Endocrine | 2 |  |  |  |  | NA | NA |
| Left anterior thigh | 4 | Progression | Endocrine | 2 |  |  |  |  | NA | NA |
| Right forearm | 4 | Progression | Skin | 1 |  |  |  |  | NA | NA |
|  | 4 | Completion of planned therapy | |  |  |  | None |  | NA | NA |
| Right upper arm | 4 | Progression | Other | 2 | Hepatic | 2 |  |  | NA | NA |
| Nov 2009 right shoulder, recurrence 2013) | 4 | Progression | Hepatic | 3 |  |  |  |  | NA | NA |
| Liver met. (oct 2009 right back) | 3 | Death |  |  |  |  | None |  | NA | NA |
| Liver met. (left leg Oct 2020) | 4 | Progression |  |  |  |  | None |  | NA | NA |
| Mets (2017 left leg) |  | Therapy stopped due to toxicity | Skin | 2 | Other | 2 | GIT | 2 | PO Prednisolone 0.5-1mg/kg/day | NA |
| Upper back | 4 | Therapy stopped due to toxicity | GIT | 2 |  |  |  |  | PO Prednisolone 0.5-1mg/kg/day | NA |
| Right foot | 4 | Progression |  |  |  |  | None |  | NA | NA |
| Lower back | 4 | Therapy stopped due to toxicity | Endocrine | 2 | GIT | 2 |  |  | NA | NA |
| Lung met. | 4 | Progression | Neuro. | 2 | GIT | 3 |  |  | IV Methylprednisolone 1-2mg/kg/day | No |
| Groin dissection (March 2015 left popliteal fossa) | 3 | Progression | Hepatic |  | Endocrine |  |  |  | NA | NA |
| Right back | 4 | Completion of planned therapy | |  |  |  | None |  | NA | NA |
| Right shoulder |  | Progression | MSK | 2 |  |  |  |  | NA | NA |
| Right big toe | 3c | Progression | Hepatic | 2 |  |  |  |  | NA | NA |
| Unknown primary | 4 | Therapy stopped due to toxicity | MSK | 2 | GIT | 2 |  |  | PO Prednisolone FLAT dose | NA |
| Lung lesion (Oct 2016 mucosal melanoma jaw) | 4 | Progression |  |  |  |  | None |  | NA | NA |
| Right neck | 4 | Completion of planned therapy | GIT | 2 |  |  |  |  | PO Prednisolone FLAT dose | NA |
| Breast | 4 | Progression | Skin | 1 | Endocrine | 3 |  |  | NA | NA |
| Unknown primary | 4 | Progression |  |  |  |  | None |  | NA | NA |
| Right groin | 4 | Progression | Renal | 2 |  |  |  |  | NA | NA |
| Neck | 4 | Therapy stopped due to toxicity | Other |  |  |  |  |  | NA | NA |
| Back (Jan 2012 back) | 4 | Progression | GIT | 2 |  |  |  |  | PO Prednisolone 0.5-1mg/kg/day | NA |
| Left groin | 4 | Progression |  |  |  |  | None |  | NA | NA |
| Ankle | 4 | Therapy stopped due to toxicity | MSK |  |  |  |  |  | NA | NA |
| Brain met. (2018 neck) | 4 | Therapy stopped due to toxicity | Hepatic | 1 | Respiratory | 3 |  |  | NA | NA |
| Abdominal met. (2013 right lower back) | 4 |  |  |  |  |  | None |  | NA | NA |
| Lung met. (2012 left neck melanoma) | 4 | Therapy stopped due to toxicity | GIT | 2 |  |  |  |  | IV Methylprednisolone 1-2mg/kg/day | No |
| Right thigh | 4 | Progression | MSK | 2 |  |  |  |  | NA | No |
| Right axilla | 4 | Progression |  |  |  |  | None |  | NA | No |
| Left ear | 4 | Completion of planned therapy | Endocrine | 2 |  |  |  |  | NA | No |
| Right shoulder | 4 | Therapy stopped due to toxicity | Skin | 3 | Hepatic | 3 | MSK | 2 | NA | No |
| Right foot | 4 | Progression | GIT | 2 |  |  |  |  | PO Prednisolone 0.5-1mg/kg/day | No |
| Left heel | 4 | Therapy stopped due to toxicity | Hepatic |  |  |  |  |  | NA | No |
| Scalp | 4 | Completion of planned therapy | |  |  |  | None |  | NA | No |
| Right temple | 3 | Therapy stopped due to toxicity | Endocrine | 2 | Hepatic | 4 |  |  | NA | No |
| Left hand | 4 | Progression |  |  |  |  | None |  |  | No |
| Ocular | 4 | Progression | GIT | 4 | Endocrine | 2 | None |  | IV Methylprednisolone 1-2mg/kg/day | Yes |
| Back | 4 | Progression |  |  |  |  | None |  | NA | No |
| Liver and lung met. (ocular melanoma 20 years ago) | 4 | Progression |  |  |  |  | None |  | NA | No |
| Right basal scalp | 4 | Completion of planned therapy | |  |  |  | None |  | NA | No |
| Left neck | 3 | Completion of planned therapy | |  |  |  | None |  | NA | No |
| Left neck | 4 | Progression | Endocrine | 2 |  | NA | None |  | NA | No |
| Left shoulder | 4 | Progression |  | NA |  | NA |  |  | NA | No |
| Multiple subcutaneous soft-tissue nodules, intramuscular deposits, probable lung metastasis, hilar lymph nodes and uptake around the heart | 4 | Therapy stopped due to toxicity | GIT | 3 |  | NA | None |  | IV Methylprednisolone 1-2mg/kg/day | Yes |
| Right back (excised) March 2016, met. March 2017 - multiple liver metastases, bone metastasis and lung metastasis | 4 | Therapy stopped due to toxicity | GIT | 2 |  |  |  |  | PO Prednisolone FLAT dose | No |
| Left cheek | 4 | Therapy stopped due to toxicity | Endocrine | 3 | GIT | 3 | Respiratory | 4 | IV Methylprednisolone 1-2mg/kg/day | No |
| Left foot |  | Progression |  |  |  |  |  |  | NA | No |
| Chest wall | 3c | Progression |  |  |  |  |  |  | NA | No |
| Widespread large volume | 4 | Progression | Skin | 1 |  |  |  |  | Topical steroid | No |

Abbreviations: GIT: Gastrointestinal; ICI: Immune checkpoint inhibitor; IrAE: Immune-related adverse events; IV: Intravenous; Met.: Metastasis; MSK: Musculoskeletal; NA: Not-applicable; Neuro.: Neurological; PO: Peroral;
